# The Role of Hypoxia on the Trimethylation of H3K27 in Podocytes

**DOI:** 10.3390/biomedicines11092475

**Published:** 2023-09-07

**Authors:** Johanna Barth, Ivonne Loeffler, Tzvetanka Bondeva, Marita Liebisch, Gunter Wolf

**Affiliations:** Department of Internal Medicine III, University Hospital Jena, Am Klinikum 1, 07747 Jena, Germany; johanna.barth@uni-jena.de (J.B.); ivonne.loeffler@med.uni-jena.de (I.L.); tzvetanka.bondeva@med.uni-jena.de (T.B.); gunter.wolf@med.uni-jena.de (G.W.)

**Keywords:** hypoxia, podocyte, H3K27me3, epigenetic, NIPP1, nuclear inhibitor of protein phosphatase 1, EZH2, enhancer of zeste homolog 2

## Abstract

Epigenetic alterations contribute to the pathogenesis of chronic diseases such as diabetes mellitus. Previous studies of our group showed that diabetic conditions reduce the trimethylation of H3K27 in podocytes in a NIPP1- (nuclear inhibitor of protein phosphatase 1) and EZH2- (enhancer of zeste homolog 2) dependent manner. It has been previously reported that in differentiated podocytes, hypoxia decreases the expression of slit diaphragm proteins and promotes foot process effacement, thereby contributing to the progression of renal disease. The exact mechanisms are, however, not completely understood. The aim of this study was to analyze the role of hypoxia and HIFs (hypoxia-inducible factor) on epigenetic changes in podocytes affecting NIPP1, EZH2 and H3K27me3, in vitro and in vivo. In vivo studies were performed with mice exposed to 10% systemic hypoxia for 3 days or injected with 3,4-DHB (dihydroxybenzoate), a PHD (prolyl hydroxylase) inhibitor, 24 h prior analyses. Immunodetection of H3K27me3, NIPP1 and EZH2 in glomerular podocytes revealed, to the best of our knowledge for the first time, that hypoxic conditions and pharmacological HIFs activation significantly reduce the expression of NIPP1 and EZH2 and diminish H3K27 trimethylation. These findings are also supported by in vitro studies using murine-differentiated podocytes.

## 1. Introduction

Chronic kidney disease (CKD) affects more than 10% of the general population worldwide [1]. Diabetic kidney disease (DKD) is the most common cause of CKD and end-stage renal disease (ESRD) that occurs in 30–40% of diabetic patients [2]. Podocyte dysfunction is a major factor in the development of DKD and correlates with proteinuria [2]. Laser scanning microscopy analyses of podocyte architecture in type 2 diabetic patients with microalbuminuria showed markers of injury, including podocyte hypertrophy, diffuse foot process effacement, as well as sites of initial detachment from the basement membrane; and in patients with late stages of proteinuria, podocyte loss and extensively denuded glomerular basement membranes [3]. Animal models revealed that extensive podocyte loss causes irreversible glomerular damage and progresses to tubulointerstitial fibrosis and ESRD [2,4,5,6].

Epigenetic changes mean variations in gene expression induced by environmental factors without variation in the DNA nucleotide sequence, such as histone methylation. EZH2 (enhancer of zeste homolog 2) catalyzes the trimethylation of lysine 27 of histone 3 (H3K27) [7]. Major clinical trials of patients with diabetes mellitus showed an increased development of DKD caused by a relatively short period of poor glucose control many years ago despite an excellent glycemic control thereafter. This phenomenon is called metabolic memory [2].

Advanced glycated end-products (AGEs) are characteristic hallmarks of DKD [8]. Previously, we have shown that AGEs significantly reduced NIPP1 and EZH2 in podocytes in in vitro conditions, as well as in diabetic mice [9,10]. We also reported that NIPP1 interacts with the methyltransferase EZH2 in podocytes [10]. Moreover, EZH2-mediated H3K27 trimethylation was also decreased in podocytes and in diabetic mice studies [10]. These data demonstrated that NIPP1 and EZH2 take part in the establishment of glycemic memory in podocytes under diabetic conditions [10]. Several published studies revealed the importance of epigenetic changes that drive the pathophysiology of DKD [11,12]. Next to changes in histone methylation, the dysregulation of DNA methylases and different miRNAs, as well as acetylation and deacetylation of histones, contribute to the progression of disease. The role of epigenetic changes in podocyte injury was also shown for histone deacetylases [13,14,15,16], Krüppel-like Factor 4 [17,18] or Wilms Tumor-1 [19].

Another pathophysiological characteristic of CKD is hypoxia, caused by numerous factors [20]. It occurs from a mismatch between oxygen demand and supply. In DKD, diabetes and hyperglycemia increase the energy consumption of tubular cells, whereas oxygen delivery is inhibited due to multiple factors [21]. Moreover, glomerular hypoxia also occurs [20]. Numerous animal and human studies emphasize the significance of hypoxia in DKD [22]. Hypoxia-inducible factors (HIFs) have been shown to be activated in renal diseases and play a central role in the cellular adaptation to hypoxia. HIFs are composed of two subunits. The β-subunit is constitutively expressed, while the α-subunit is regulated by molecular oxygen. Under normoxic conditions, HIF-α is degraded after hydroxylation by prolyl hydroxylase domain (PHD) proteins and subsequently ubiquitinated by the E3 ubiquitin ligase activity of the von Hippel-Lindau tumor suppressor protein (pVHL), thereby targeting HIF-α for degradation by the proteasome [23]. Hypoxia stabilizes HIF-α by inhibition of prolyl hydroxylation. The α-subunit translocalizes to the nucleus, where it heterodimerizes with the β-subunit. The HIF heterodimer initiates the expression of various genes involved in hypoxia adaption [22,24]. In podocytes, HIFs induce various complications like cytoskeletal derangement, foot process effacement or slit diaphragm dysfunction [24,25,26,27,28,29,30,31].

The role of hypoxia on epigenetic changes in CKD has not been well studied, especially not in podocytes. Lin et al. [32] described changes in the expression of three lncRNAs under hypoxic and inflammatory conditions in human proximal tubular cells [32]. Kroening et al. [33] showed that hypoxia reduces the expression of CTGF (connective tissue growth factor, a stimulator of extra cellular matrix) in human kidney epithelial cells with the involvement of DNA methylation [33]. Some publications describe a so-called hypoxic memory that drives the transition of acute kidney injury (AKI) to CKDs [34,35]. Epidemiological studies showed that even after AKI, a certain percentage of patients who recovered from AKI develop CKD [34,35]. Therefore, renal hypoxia can also induce the establishment of cellular memory.

Taking all this into consideration, we aimed to analyze whether hypoxic conditions in podocytes and in mice can induce epigenetic changes through NIPP1 and EZH2-mediated H3K27me3.

## 2. Materials and Methods

### 2.1. Podocyte Cell Culture

For the in vitro analyses, we used a podocyte cell line isolated from mouse (E11, CLS Cell Lines Service GmbH, Eppelheim, Germany) [36]. Undifferentiated podocytes were grown in RPMI 1640 medium (Invitrogen, Schwerte, Germany) supplemented with 10% heat-inactivated FCS (Gibco, Darmstadt, Germany) and 10 U/mL mouse γ-interferon (Invigate, Jena, Germany) at 33 °C in a 5% CO_2_ atmosphere (permissive conditions). For differentiation of these cells, the temperature was increased to 37 °C and γ-interferon was removed for at least 2 weeks (nonpermissive conditions). The differentiated mouse podocytes were used in all in vitro cell culture experiments and termed only podocytes throughout the manuscript.

For induction of hypoxia, murine podocytes were cultured in a cell incubator at 10% oxygen for 24 h.

### 2.2. Reverse Transcription and Real-Time PCR

Isolation of total RNA, cDNA synthesis and RT-PCR were carried out as previously described [10]. Briefly, mRNA was isolated using the RNeasy Mini kit (Qiagen, Hilden, Germany) according to the manufacture’s instructions. For cDNA synthesis, the Reverse Transcription System (Promega Corporation, Madison, WI, USA) was used. The primer sequences used for Real-Time PCR were published elsewhere [10]. The expressions of NIPP1 and EZH2 and the housekeeping gene (HPRT-hypoxanthine-guanine phosphoribosyltransferase) were simultaneously analyzed. Quantification was performed using the ∆∆C_T_ method [37]. Briefly, C_T_ values of NIPP1 and EZH2 were normalized to the C_T_ values of HPRT. The ratio of the gene expression (R = 2^(−∆∆CT)^) was normalized to the mean value of the control normoxic cells.

### 2.3. Western Blot Analysis

Isolation of whole cell protein was performed using complete lysis-M reagent (Roche, Mannheim, Germany) according to the manufacturer’s instructions. SDS-PAGE and Western blot assay were carried out as described [10]. For the blocking step, PVDF membrane was incubated with ROTI^®^Block (Carl Roth GmbH, Karlsruhe, Germany) for 1 h at room temperature. The primary antibody was incubated overnight at 4 °C. After three washing steps PVDF membrane was incubated with the according secondary antibody for 1 h at room temperature. The following primary antibodies were used: polyclonal goat anti-NIPP1 (aviva systems biology, San Diego, CA, USA), polyclonal rabbit anti-EZH2 (active motif, Regensburg, Germany), polyclonal rabbit anti-H3K27me3 (GeneTex, Alton Pkwy Irvine, CA, USA), monoclonal mouse anti-TATA box-binding protein (TBP; NSJ bioreagents, San Diago, CA, USA). The following HRP (horse radish peroxidase)-coupled secondary antibodies were used: rabbit anti-goat IgG, goat anti-mouse IgG, goat anti-rabbit IgG (all purchased KPL, Gaithersburg, MA, USA).

Protein expression was visualized using ECL reagent (enhanced chemiluminescence; PerkinElmer, Rodgau, Germany) and the G:Box chemi XX6 detection system (Syngene, Cambridge, UK).

The protein expression was quantified using ImageJ software (NIH, Bethesda, Rockville, MD, USA). The ratio of the expression of the protein of interest was normalized to the housekeeping protein TBP and was calculated to the mean value of the control normoxic cells.

### 2.4. Immunofluorescence Staining of Cultured Differentiated Podocytes

Differentiated podocytes were cultured in chamber slides (Permanox; Thermo Scientific, Nunc, Rochester, NY, USA). After the incubation under normoxic (control) or hypoxic conditions, cells were fixed and permeabilized with ROTI-Histofix^®^ 4% formaldehyde/0.1% TritonX-100 (both Carl Roth GmbH). Fixed cells were blocked with 5% BSA (bovine serum albumin; Carl Roth GmbH) at room temperature for 1 h. Primary antibody was diluted in blocking solution and incubated overnight at 4 °C. Following primary antibodies were used: polyclonal goat anti-NIPP1 (Aviva systems biology), polyclonal rabbit anti-EZH2 (Active motif) and monoclonal rabbit anti-H3K27me3-Alexa Fluor^®^647 (Abcam, Cambridge, UK). After three washing steps and an additional blocking step for 30 min at room temperature, fixed cells were incubated with the appropriate secondary antibody. Following secondary antibodies for immunofluorescence staining: goat anti-rabbit IgG DyLight^®^594 and horse anti-goat IgG DyLight^®^594 (Vector Laboratories, Burlingame, CA, USA) were used. Nuclei were counterstained with DAPI (4′,6-diamidino-2-phenylindole; Sigma-Aldrich, Merck, Darmstadt, Germany) for 10 min at room temperature. Slides were mounted with VECTASHIELD Vibrance^®^ Antifade Mounting Medium (Vector Laboratories, Newark, CA, USA). Staining was analyzed using the Zeiss Axio Imager. Z2 and Zen 2.5 blue software (Carl Zeiss, Jena, Germany). For single cell quantification, the ImageJ software (NIH) was used. For evaluation, N experiments were carried out with n analyzed cells at each time.

### 2.5. Animal Studies

All animal experiments were approved by the Local Ethic Committee of Thueringer Landesamt fuer Verbraucherschutz and were performed in accordance with the German Animal Protection Law. Mice were housed in a pathogen-free facility with a 12 h light-dark cycle, on standard chow and water ad libitum. Two different mouse approaches were used: (1) Mice were exposed to 10% systemic hypoxia for 3 days as previously described [38]. For control, normoxic mice (21% oxygen) were used [38]. (2) For pharmacological HIFs activation mice were treated with 150 mg/kg body weight ethyl 3,4-DHB (dihydroxybenzoate) i.p., as described previously [39]. Moreover, 3,4-DHB is shown to be a non-specific PHD inhibitor for all PHDs. The control mice were injected with 0.9% NaCl i.p. Mice were sacrificed 24 h after the experimental treatment in accordance with the German Animal Protection Law [39].

### 2.6. Double-Immunofluorescence Staining of Paraffin Kidney Sections

Double-immunofluorescence stainings of paraffin kidney sections were carried out as described [10]. The two stainings were performed independently. First, the Synaptopodin-staining was made, and after that the NIPP1, respectively, EZH2 or H3K27me3 staining. For each staining, 2.5 µm paraffin kidney sections were blocked with 5% BSA (Carl Roth GmbH) for 1 h at room temperature. Afterwards, kidney sections were incubated with the primary antibody overnight at 4 °C. After three washing steps kidney sections were blocked again with 5% BSA (Carl Roth GmbH) for 30 min at room temperature. Next, secondary antibody was incubated for 1 h at room temperature. Following primary and appropriate secondary antibodies were used: polyclonal goat anti-NIPP1 (Aviva systems biology) and horse anti-goat IgG DyLight^®^594 (Vector Laboratories), or polyclonal rabbit anti-EZH2 (Active motif) and goat anti-rabbit IgG DyLight^®^594 (Vector Laboratories), or monoclonal rabbit anti-H3K27me3-Alexa Fluor^®^647 (Abcam), or monoclonal mouse anti-Synaptopodin antibody (Santa Cruz Biotechnology, Dallas, Texas, USA) and horse anti-mouse IgG DyLight^®^488 (Vector Laboratories). Nuclei were counterstained with DAPI (Sigma-Aldrich, Merck). Staining was analyzed using the Zeiss Axio Imager. Z2 and Zen 2.5 blue software (Carl Zeiss). For quantification of podocyte protein expression, the ImageJ software (NIH) was used. For evaluation, N animals with n glomeruli at each time were analyzed.

### 2.7. Statistics

The data are shown as box/whisker-dot plots, drawn using SigmaPlot 14.5 (Systat, Frankfurt am Main, Germany). The results were analyzed using the unpaired, two-tailed Students t-Test. Differences were considered significant when *p* ≤ 0.05.

## 3. Results

### 3.1. Hypoxia Affects NIPP1, EZH2 and H3K27me3 in Podocytes In Vitro

**Figure 1:**
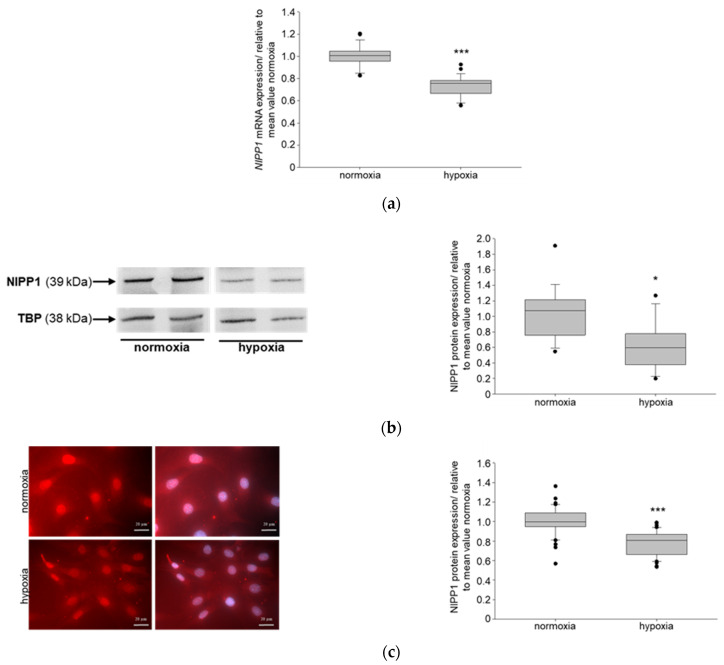
Detection of NIPP1 (nuclear inhibitor of protein phosphatase 1) expression in cultured podocytes under hypoxic conditions: (**a**) *NIPP1* mRNA expression; N = 3, n = 12; *** *p* ≤ 0.001 compared with normoxia; (**b**) Western blot of NIPP1 protein expression and quantification; TBP-TATA box binding protein; N = 5, n = 4; * *p* ≤ 0.05 compared with normoxia; (**c**) NIPP1 immunofluorescence and quantification; red-NIPP1, blue-DAPI (4′,6-diamidino-2-phenylindole), scale bar: 20 μm; N = 2, n = 10; *** *p* ≤ 0.001 compared with normoxia.

**Figure 2:**
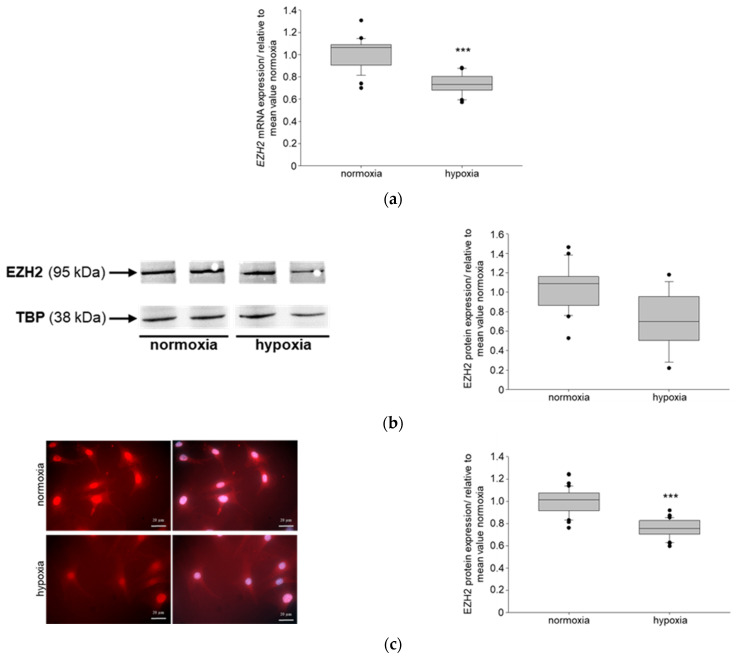
Detection of EZH2 (enhancer of zeste homolog 2) expression in cultured podocytes under hypoxic conditions: (**a**) *EZH2* mRNA expression; N = 3, n = 12; *** *p* ≤ 0.001 compared with normoxia; (**b**) Western blot of EZH2 protein expression and quantification; TBP-TATA box binding protein; N = 6, n = 4; (**c**) EZH2 immunofluorescence and quantification; red-EZH2, blue-DAPI; N = 2, n = 10, scale bar: 20 μm; *** *p* ≤ 0.001 compared with normoxia.

CKDs have a major effect on health worldwide, with vast increasing evidence. It’s primarily caused by diabetes [40]. One pathophysiological characteristic of CKD is hypoxia [20]. To study if NIPP1 and EZH2 are affected in hypoxic conditions, murine podocytes were incubated at 10% O_2_ (hypoxia), respectively, 21% O_2_ (normoxia, control) for 24 h. Real-time PCR (RT PCR) data showed that hypoxia significantly reduced the mRNA expression of both, NIPP1 (Figure 1a) and EZH2 (Figure 2a) in podocytes in vitro. Western Blot and immunofluorescence demonstrated that protein expressions of NIPP1 (Figure 1b,c) and EZH2 (Figure 2b,c) were also significantly diminished in podocytes under 10% O_2_ compared with the normoxic control.

**Figure 3:**
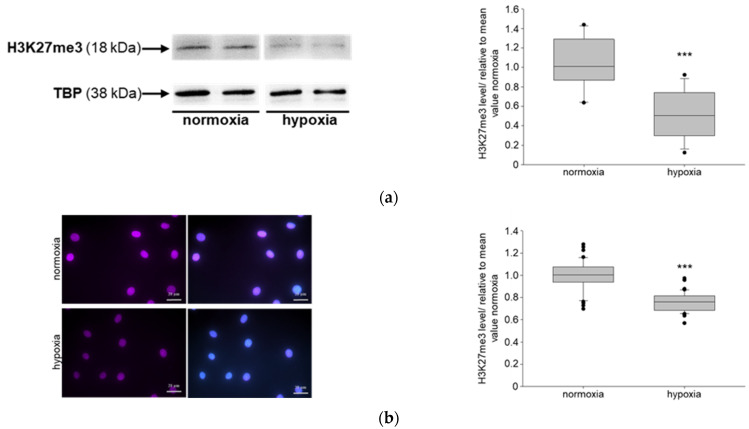
Detection of H3K27me3 in cultured podocytes under hypoxic conditions: (**a**) Western blot of H3K27me3 and quantification; TBP-TATA box binding protein; N = 4, n = 4; *** *p* ≤ 0.001 compared with normoxia; (**b**) H3K27me3 immunofluorescence and quantification; purple-H3K27me3, blue-DAPI; N = 2, n = 10, scale bar: 20 μm; *** *p* ≤ 0.001 compared with normoxia.

As shown previously, reduction in the NIPP1 and EZH2 expression by siRNA transfection affect the trimethylation of H3K27 in podocytes [10]. Therefore, we also tested whether hypoxia may influence the level of H3K27me3. Both Western blot and immunofluorescence studies clearly demonstrates that the trimethylation of H3K27 was significantly reduced in podocytes that were incubated under hypoxic conditions compared to the control cells (Figure 3).

### 3.2. Systemic Hypoxia Influences NIPP1 and EZH2 Protein Expression and Trimethylation of H3K27

We further analyzed the findings from cell culture experiments using two different mouse models of hypoxia. First, we used kidney samples from mice that were housed for 3 days in an environment with 10% O_2_, or, respectively, 21% O_2_ as control. Previous immunohistochemistry experiments of kidney paraffin sections from these mice showed an obvious increase in HIFs expression in hypoxic mice [38]. Additionally, the serum concentrations of erythropoietin (a known target gene of HIFs) were also significantly elevated in the mice that were exposed to systemic hypoxia. Furthermore, urinary albumin-to-creatinine ration (ACR), serum urea and serum creatinine (renal function parameters) were significantly increased in mice under hypoxic conditions compared to the control animals [38].

**Figure 4:**
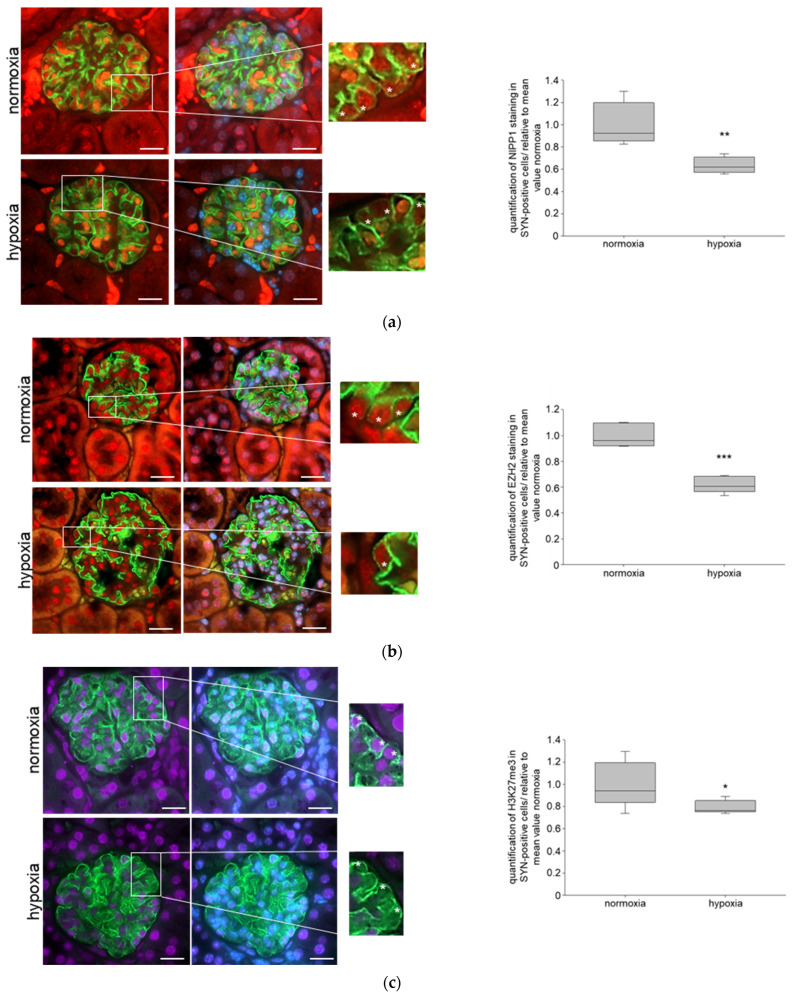
Role of systemic hypoxia (10% O_2_) on NIPP1 expression, EZH2 expression and trimethylation of H3K27 in mice: (**a**) double immunofluorescence staining of NIPP1 and Synaptopodin (SYN); red-NIPP1, green-SYN, blue-DAPI; N = 6, n = 20; representative podocytes are tagged with white asterisks; ** *p* ≤ 0.01 compared with normoxia; (**b**) double immunofluorescence staining of EZH2 and SYN; red-EZH2, green-SYN, blue-DAPI; N = 6, n = 20; representative podocytes are tagged with white asterisks; *** *p* ≤ 0.001 compared with normoxia; (**c**) double immunofluorescence staining of H3K27me3 and SYN; purple-H3K27me3, green-SYN, blue-DAPI; N = 6, n = 20; representative podocytes are labelled with white asterisks; * *p* ≤ 0.05 compared with normoxia.

To study the protein expression in podocytes of the normoxic and hypoxic mice, we prepared double-immunofluorescence analyses of paraffin kidney sections of NIPP1 or EZH2 or trimethylated H3K27 with Synaptopodin (SYN), a marker of podocytes. Quantification of NIPP1 (Figure 4a), EZH2 (Figure 4b) and H3K27me3 (Figure 4c) in SYN-positive cells showed a significant reduction under hypoxic conditions compared to the normoxic control mice.

### 3.3. Pharmacological Activation of HIFs Reduces the Expression of NIPP1 and EZH2 as Well as H3K27 Trimethlyation

**Figure 5:**
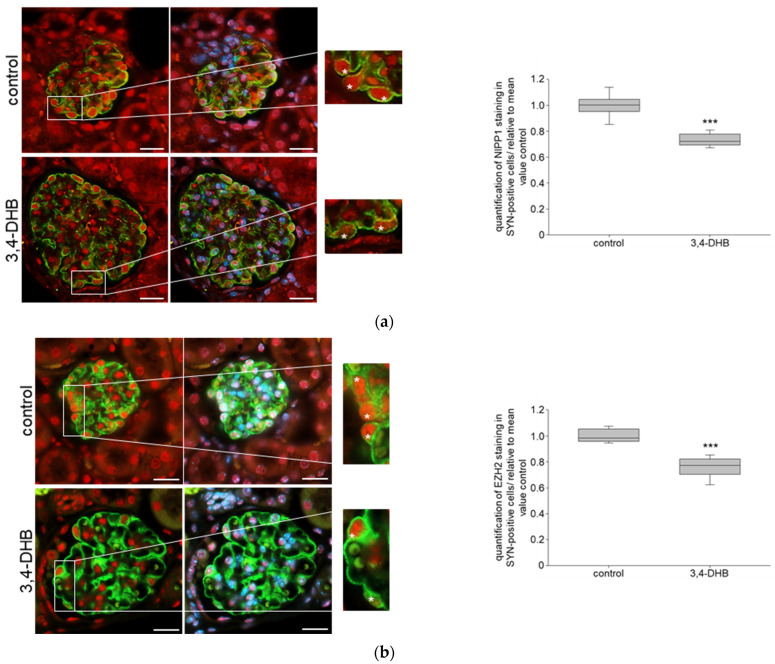

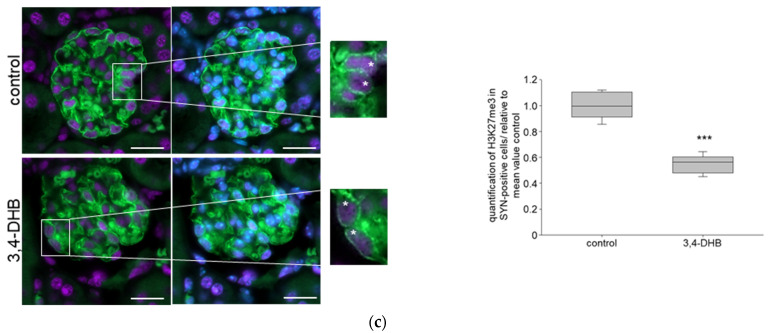
Role of pharmacological activation of HIFs on NIPP1, EZH2 and H3K27me3 in mice: (**a**) double immunofluorescence staining of NIPP1 and SYN; red-NIPP1, green-SYN, blue-DAPI; N = 6, n = 15; representative podocytes are tagged with white asterisks; 3,4-DHB—3,4-dihydroxybenzoate; *** *p* ≤ 0.001 compared with control; (**b**) double immunofluorescence staining of EZH2 and SYN; red-EZH2, green-SYN, blue-DAPI; N = 6, n = 15; representative podocytes are tagged with white asterisks; *** *p* ≤ 0.001 compared with control; (**c**) double immunofluorescence staining of H3K27me3 and SYN; purple-H3K27me3, green-SYN, blue-DAPI; N = 6, n = 15; representative podocytes are tagged with white asterisks; *** *p* ≤ 0.001 compared with control.

HIFs play a central role in hypoxia and target hundreds of genes that are important for tissue adaption [23]. We performed an in vivo study where mice were treated with 3,4-DHB (dihydroxybenzoate), an unspecific inhibitor of prolyl hydroxylases and, consequently, an activator of HIFs. We have previously shown using immunohistochemistry detection of HIFs in renal sections that the 3,4-DHB treatment induces HIFs accumulation compared with the control mice [39]. Moreover, due to HIFs activation plasma VEGF (vascular endothelial growth factor, a known HIFs target gene) and plasma-erythropoietin were also increased. While there were no significant differences in urinary ACR, the urinary concentration of KIM-1 (kidney injury molecule), an early renal injury marker, was elevated in the 3,4-DHB-treated mice [39]. We further studied the protein expression of NIPP1 and EZH2 as well as H3K27me3 in podocytes of 3,4-DHB-treated mice and performed double-immunofluorescence analyses of paraffin kidney sections with SYN. The results showed that pharmacological activation of HIFs significantly reduced the expression of both NIPP1 (Figure 5a) and EZH2 (Figure 5b) in glomerular podocytes. Additionally, the trimethylation of H3K27 was also significantly diminished (Figure 5c).

## 4. Discussion

The role of hypoxia on epigenetic changes in podocytes in CKD has been only fragmentally investigated. Because epigenetic changes occur in many diseases, epigenetics can be used as preventive, diagnostic, and therapeutic markers. This underlines the importance of research in this field. Our studies showed, to the best of our knowledge, for the first time, that hypoxic conditions change trimethylation of H3K27 in podocytes in vitro and in vivo in mice. Both systemic hypoxia and pharmacological activation of HIFs via the application of 3,4-DHB in mice lead to a significantly reduced H3K27me3 in glomerular podocytes. Therefore, our data strongly suggest that hypoxia plays a likely role in epigenetic H3K27me3 variation in podocytes, as previously reported for AGEs [10]. This suggests that hypoxia can also contribute to an epigenetic memory in podocytes in CKD. This has to be analyzed in more detail.

There are some limited studies describing the role of changes in H3K27 trimethylation in the pathophysiology of podocyte injury. In agreement with our data, Majumder et al. [41] analyzed podocytes from patients with focal segmental glomerulosclerosis or DKD and found reduced H3K27me3 [41]. Siddiqi et al. [42] showed that depletion of EZH2 and, consequently, reduced H3K27me3 in diabetic rats induces podocyte injury, oxidative stress and proteinuria [42]. Other findings describe an overall reduced H3K27 trimethylation in the kidneys of high-fat-fed rats which goes along with the metabolic memory [43], in diabetic mouse models [44,45,46,47,48] or in nephrotic patients [49,50]. However, there are also conflicting studies. Two reports show an increase in H3K27me3 in podocytes stimulated with high glucose, which was associated with podocyte damage [51,52]. Controversial data might be due to the use of different experimental approaches and kidney injury models. For example, Zhou et al. [53] describe an increased EZH2 expression and trimethylation of H3K27 using a mouse model of unilateral ureteral obstruction disease [53].

Because the trimethylation of H3K27 is catalyzed by EZH2, we also analyzed its expression under hypoxic conditions. Our results showed that hypoxia significantly reduced the expression of EZH2 in podocytes in vitro and in vivo in mice. Additionally, pharmacological activation of HIFs also suppresses EZH2 expression in podocytes in mice. Various studies show the importance of EZH2 in hypoxia [54,55]. It was reported that reactive oxygen species could facilitate the binding of HIF-1α to the EZH2 promotor. Liu et al. [56] depict that the upregulation of EZH2 in a renal ischemia/reperfusion (I/R) injury model activates oxidative stress and hypoxia in mice [56]. It might seem conflicting with our results at first sight, but the I/R model stands for acute kidney injury. In addition, for in vitro analyses, they used 1% oxygen, very unphysiological conditions for cell culture hypoxia experiments likely inducing apoptosis and cell death. Jia et al. [57] showed that TGF-β suppresses the expression of EZH2 and, consequently, trimethylation of H3K27 on known TGF-β target genes in kidney mesangial cells, which mediates nephropathy [57].

Previously, studies from our group showed that NIPP1 and EZH2 interact in podocytes, and both EZH2 and NIPP1 siRNA transfection reduce H3K27me3 [10]. Thus, we also analyzed the expression of NIPP1 under hypoxic conditions. The data shows that hypoxia in vitro and in vivo in mice, as well as pharmacological activation of HIFs in mice, significantly reduced NIPP1 expression in podocytes. Some studies address the role of NIPP1 in hypoxia [58,59,60], but until now, not in renal cells.

**Figure 6:**
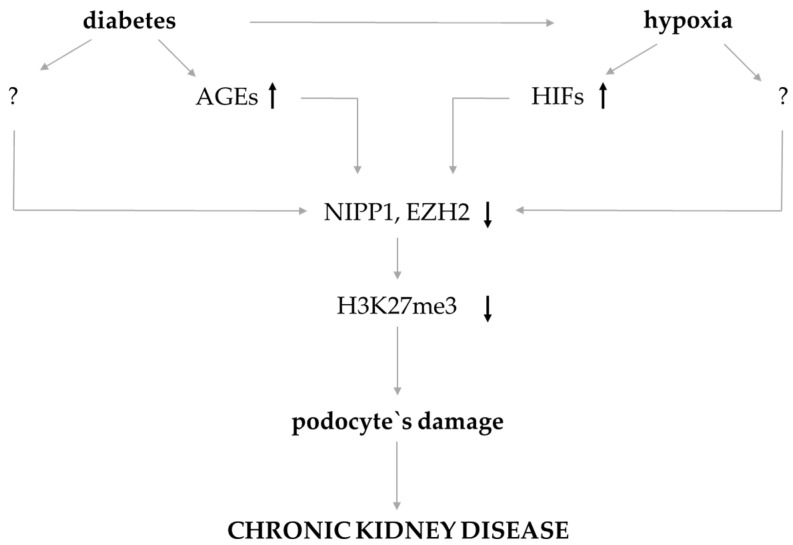
Schematic illustration of the assumed mechanism that contributes to epigenetic changes of H3K27me3 and podocyte injury in CKD.

Our data demonstrated that both AGEs/diabetes [9,10] and hypoxia cause a reduction in NIPP1 and EZH2 expression in podocytes, which goes along with lower H3K27me3 levels. We could show that siRNA-mediated inhibition of NIPP1 or EZH2 induces the expression of injury-related molecules in podocytes, including p27^Kip1^, RAGE (receptor of advanced glycation end-products), Snail or TGF-β1 [9,10]; and for NIPP1, NIPP1 siRNA transfection induces podocyte hypertrophy [9]. From the results presented here, we concluded that hypoxia also induces or contributes to podocyte injury seen in CKD in a manner illustrated in Figure 6. We would not exclude that besides HIFs there are other mediators induced by hypoxia that inhibit NIPP1 and/or EZH2 expression as well as a reduction in H3K27me3. Additionally, next to methyltransferases, demethylases also play an important role in histone modifications, which is not considered in this study. It is important to learn more about the function of epigenetics on the pathophysiology of podocyte injury. Therefore, it is necessary to address the molecular mechanisms of hypoxia-dependent NIPP1 and EZH2 suppression in more detail in future analysis.

## Data Availability

The data presented in this study are available on request from the corresponding author.
